# Simple and green fabrication of an Ag-Ag_2_S/TiO_2_/cellulose biocomposite film with enhanced photocatalytic and antibacterial activity

**DOI:** 10.1039/d5ra04993h

**Published:** 2025-09-19

**Authors:** Mouheb Sboui, Khalid A. Alamry, Youssef O. Al-Ghamdi, Balachandran Subramanian, Meenakshisundaram Swaminathan, Mahmoud A. Hussein

**Affiliations:** a Department of Chemistry, Faculty of Sciences, University of Sfax Sfax BP1171-3018 Tunisia sboui.mouheb@gmail.com; b Chemistry Department, Faculty of Science, King Abdulaziz University P. O. Box 80203 Jeddah 21589 Saudi Arabia; c Department of Chemistry, College of Science Al-Zulfi, Majmaah University Al-Majmaah 11952 Saudi Arabia; d Functional Materials and Materials Chemistry Laboratory, Saveetha Dental College and Hospitals, Saveetha Institute of Medical and Technical Sciences, Saveetha University Chennai 600 077 Tamil Nadu India; e Nanomaterials Laboratory, Department of Chemistry, International Research Centre, Kalasalingam Academy of Research and Education Krishnankoil 626126 India; f Chemistry Department, Faculty of Science, Assiut University Assiut 71516 Egypt

## Abstract

Cellulose-based biocomposites exhibit significant potential and versatility in advanced photocatalyst development for environmental remediation. In this study, we synthesized an Ag-Ag_2_S/TiO_2_/CT (cotton textile) biocomposite film as an efficient hybrid photocatalyst through a straightforward hydrothermal process to create TiO_2_, followed by a dipping process in an Ag-Ag_2_S solution. This biocomposite has shown a high decomposition efficiency of 97% towards *o*-toluidine as a carcinogenic compound in aqueous media after 6 h of light irradiation and complete oxidation of alcoholic compounds (which include 1-butanol, 1-propanol, and ethanol) as volatile organic compounds (VOCs) in a gaseous medium under sunlight at room temperature. Moreover, it showed notable antibacterial activity against *Escherichia coli*. This high efficiency of Ag-Ag_2_S/TiO_2_/CT can be, in particular, due to the synergistic effect between Ag-Ag_2_S and TiO_2_, which includes the smooth transfer of charges owing to their efficient separation, recombination inhibition, and improved absorption of visible light. Moreover, the prepared hybrid photocatalyst showed enhanced mechanical strength, high stability and strong durability, ensuring its reuse for several cycles. Our findings indicate that Ag-Ag_2_S/TiO_2_/CT holds promise as a multifunctional material for water treatment and is capable of removing organic pollutants, eliminating bacteria, and purifying air simultaneously.

## Abstract 2

Cellulose-based biocomposites exhibit significant potential and versatility in advanced photocatalyst development for environmental remediation.

## Graphical abstract

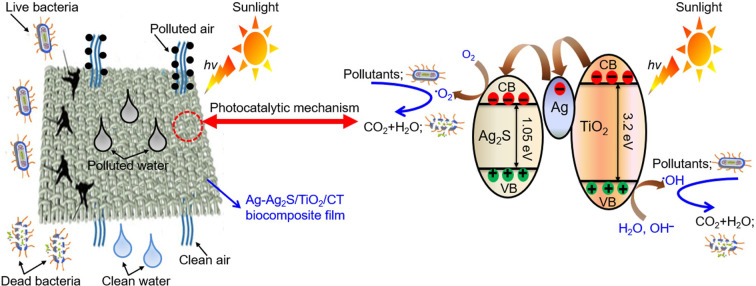

## 1. Introduction

In the past years, the textile industry has witnessed great interest in the surface functionalization of textiles with semiconductor photocatalysts to produce new fabrics with unique chemical and physical properties.^1–7^ Through this path, many new properties can be included in textiles, such as ultraviolet (UV) protection, flame retardancy, anti-smell, self-cleaning, and antimicrobial properties.^4–9^ Currently, photocatalyst-based self-cleaning textiles are used in many environmental and biomedical applications, such as air purification, treatment of undrinkable water, and microbial killing.^4,6,10–13^ This may be because it is an efficient, clean and inexpensive technology^4,6,7^ that can have many other advantages: (i) easy separation of the photocatalyst from the photocatalytic reaction since it is immobilized on the surface of a textile; (ii) ease of recovery compared to photocatalysts in powder form requiring a filtration process for recovery; and (iii) the possibility of recycling photocatalyst particles.^4,6,7^ In general, two different methods are utilized in the production of self-cleaning textiles. The first approach is mainly based on the preparation of superhydrophobic surfaces for the sliding of water droplets containing contaminants and their easy rolling.^14^ The second approach is based on a hydrophilic property, which exploits photocatalysis to decompose pollutants. By exposing textiles modified by a semiconductor photocatalyst to light, molecules of contaminants can be chemically degraded into simpler species, such as H_2_O and CO_2_.^4,15^

In this context, TiO_2_ is one of the most promising photocatalysts owing to its effectiveness in treating wastewater and efficiently purifying the air of volatile organic compounds (VOCs), in addition to its low cost, non-toxicity, and stability.^4,6,7,11–13,16,17^ Despite these unique features and characteristics of TiO_2_, its application still encounters some difficulties due to its large band gap, which needs high-energy UV irradiation for activation, as well as the recombination of its photogenerated charges.^4,12,18^ For this reason, several approaches have been adopted to improve TiO_2_ activity under visible light,^4,10–12,19^ and among them, the coupling of TiO_2_ with other narrow-band photocatalysts can be an effective approach to producing photocatalysts that respond to visible light.^4,9,11,12^ In this regard, the Ag-Ag_2_S plasmonic photocatalyst has inspired the interest of many researchers owing to its high performance,^20–26^ making it a potential candidate to be associated with TiO_2_.^25–29^ Ag-Ag_2_S is composed of silver sulfide (Ag_2_S), which has several unique properties such as non-toxicity, high efficiency in removing organic pollutants, and a narrow band gap (∼1.1 eV) that makes it capable of absorbing a wide solar spectrum.^30,31^ Ag_2_S has been utilized in a number of applications as solar cells,^32,33^ the production of hydrogen,^34–36^ and the photocatalytic degradation of organic pollutants.^37,38^ Ag_2_S has also been used as a photocatalyst for drinking water disinfection and wastewater treatment,^39,40^ and as a photoelectrode.^41–43^ Ag metal (Ag^0^) has been added to Ag_2_S to enhance its photoelectrochemical and photocatalytic properties, as Ag^0^ helps suppress charge recombination and enhance photocatalyst efficiency.^20–22,25,26^ Ag-Ag_2_S has been used in several applications, including the reduction of heavy ions, degradation of organic dyes, antibacterial agents, Li/Na ion battery anodes, and as an efficient catalyst for hydrogen evolution.^20,23,24,44–49^ However, it remains unknown whether Ag-Ag_2_S can be utilized as an efficient and stable photocatalyst towards the decomposition of VOCs in the gaseous phase.

Herein, we report a new pathway for the preparation of a multifunctional photoactive textile based on TiO_2_ with high performance driven by sunlight. For this purpose, TiO_2_ coupled with Ag-Ag_2_S was prepared as a visible-light-sensitive plasmonic photocatalyst immobilized on cotton textile (CT) using a moderate hydrothermal process to produce TiO_2_/CT, followed by dipping in Ag-Ag_2_S solution. The efficiency of the photoactive textiles was evaluated by the decomposition of *o*-toluidine (TOD) in an aqueous medium, and alcoholic compounds (ethanol, 1-propanol, and 1-butanol) in the gaseous phase under simulated sunlight irradiation. Their antibacterial properties have also been verified by using *Escherichia coli* (*E. coli*) as a model of pathogenic bacteria. The mechanical strength, durability, and reuse of prepared textiles were also verified. According to the literature, there have been no reports on Ag-Ag_2_S/TiO_2_/CT as a hybrid multifunctional photocatalyst or on air treatment of VOCs using Ag-Ag_2_S/TiO_2_.

## 2. Experimental

### 2.1. Synthesis of Ag-Ag_2_S/TiO_2_/CT biocomposite films

Ag-Ag_2_S/TiO_2_/CT biocomposite films were synthesized through a straightforward hydrothermal process to first prepare TiO_2_/CT, followed by a dipping process of TiO_2_/CT in Ag-Ag_2_S solution, which produced Ag-Ag_2_S/TiO_2_/CT by photoreduction. More information is provided in the SI.

### 2.2. Characterization of photoactive textiles

The physicochemical, morphological, and optical properties of the prepared textiles have been verified by several techniques. More information is provided in the SI.

### 2.3. Photocatalytic experiments

#### 2.3.1. Photocatalytic experiments in the liquid phase

The efficiency of Ag-Ag_2_S/TiO_2_/CT in wastewater treatment was studied *via* the use of *o*-toluidine (TOD) as a model for carcinogenic compounds. All TOD photodegradation tests were performed under the same conditions. More information is provided in the SI.

#### 2.3.2. Photocatalytic experiments in the gas phase

Three gaseous alcoholic compounds (1-butanol, 1-propanol, and ethanol) were selected as VOC models to study the efficacy of Ag-Ag_2_S/TiO_2_/CT in the purification of air under simulated sunlight irradiation under ambient conditions. More information is provided in the SI.

#### 2.3.3. Antibacterial experiments

*E. coli* (ATCC 25922) was used as an experimental organism under light and dark conditions to study the efficacy of Ag-Ag_2_S/TiO_2_/CT in the photocatalytic disinfection of bacteria. More information is provided in the SI.

## 3. Results and discussion

### 3.1. XRD analysis

**Fig. 1:**
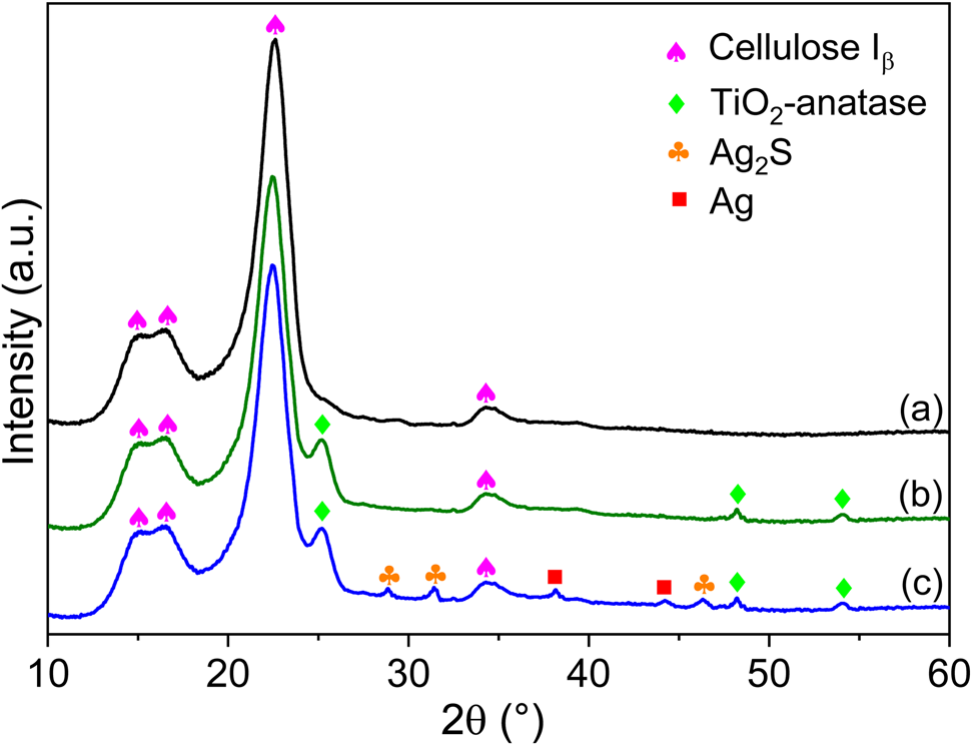
X-ray diffractogram of pristine CT (a), TiO_2_/CT (b), and Ag-Ag_2_S/TiO_2_/CT (c).

XRD was utilized to verify the crystal structure of the textile before and after modification (Fig. 1). The pristine textile showed four distinct diffraction peaks of cellulose *I*_β_ at 14.9°, 16.6°, 22.65°, and 34.4° attributed to the (1−10), (110), (200), and (004) crystalline planes, respectively.^50^ After being modified with TiO_2_ (Fig. 1b), three new peaks appeared alongside the peaks of cellulose *I*_β_. These peaks are located at 25.2°, 48.1°, and 54°, attributed to the (101), (200), and (105) plane reflections of anatase TiO_2_ (ICDD no. 21-1272).^51^ For Ag-Ag_2_S/TiO_2_/CT (Fig. 1c), new peaks attributed to Ag and Ag_2_S have emerged alongside the peaks of TiO_2_ and cellulose *I*_β_. Ag_2_S peaks observed at 28.9°, 31.4°, and 46.4° were attributed to (111), (−112), and (−123), respectively (ICDD no. 14-0072).^39,52,53^ The peaks of the Ag seen at 38.2° and 44.4° were attributed to (111) and (200), respectively (ICDD no. 65-2871).^26,52^ In the XRD pattern of Ag-Ag_2_S/TiO_2_/CT, no peaks other than the peaks of cellulose *I*_β_, TiO_2_ anatase, Ag, and Ag_2_S were observed, confirming the purity of the crystal structure of the prepared textiles. The cellulose *I*_β_ peaks did not disappear or change after functionalization of the textile with TiO_2_ and Ag-Ag_2_S, which indicates that the textile retains its structure, thanks to the functionalization method used in this work.

### 3.2. Raman analysis

**Fig. 2:**
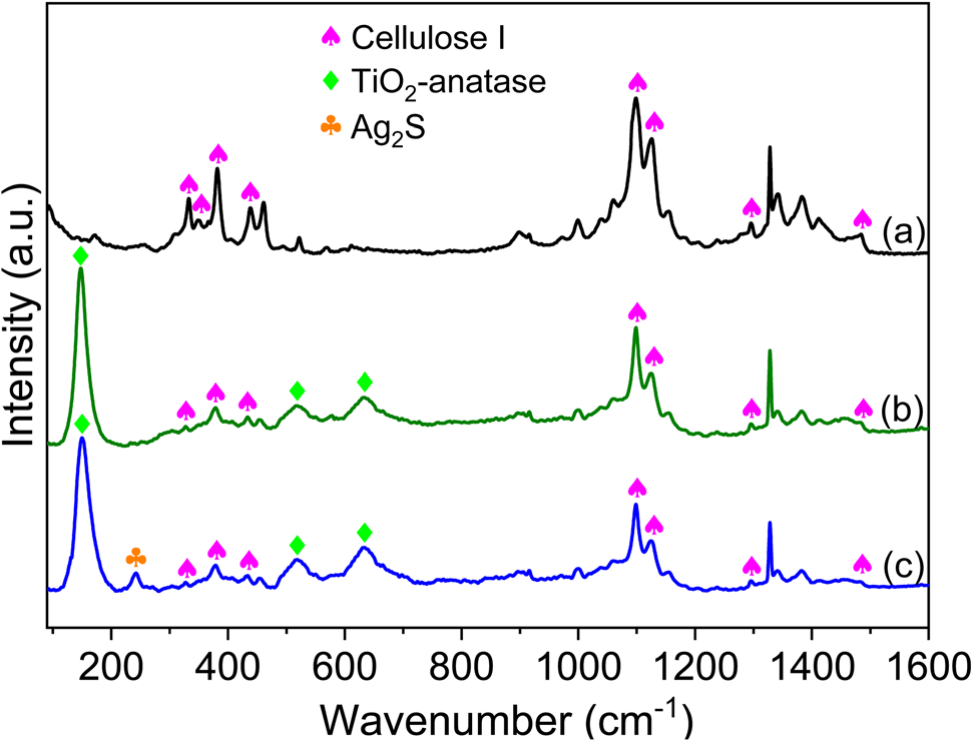
Raman spectra of pristine CT (a), TiO_2_/CT (b), and Ag-Ag_2_S/TiO_2_/CT (c).

Raman spectroscopy surpasses XRD by being somewhat more sensitive to TiO_2_ particles with low content on the surface of textiles.^54^ Therefore, the textile samples were analyzed by Raman measurements in order to further verify their structural characteristics (Fig. 2). The unmodified textile shows perfect compatibility with cellulose I as stated in the literature.^16,54–57^ After the textile was modified with TiO_2_ (Fig. 2b), new peaks were revealed along with the bands of cellulose I. These peaks were observed at 147, 517, and 639 cm^−1^, and are related to TiO_2_ anatase.^16,54,56,57^ It should be noted that no other peaks were detected in the TiO_2_/CT spectrum other than those related to TiO_2_ anatase, proving the purity of its crystalline structure. For Ag-Ag_2_S/TiO_2_/CT (Fig. 2c), both cellulose I and TiO_2_ peaks were observed along with an additional peak at 241 cm^−1^, which is related to Ag_2_S NPs,^58–60^ proving the successful functionalization of the textile with both Ag-Ag_2_S and TiO_2_.

### 3.3. FE-SEM analysis

**Fig. 3:**
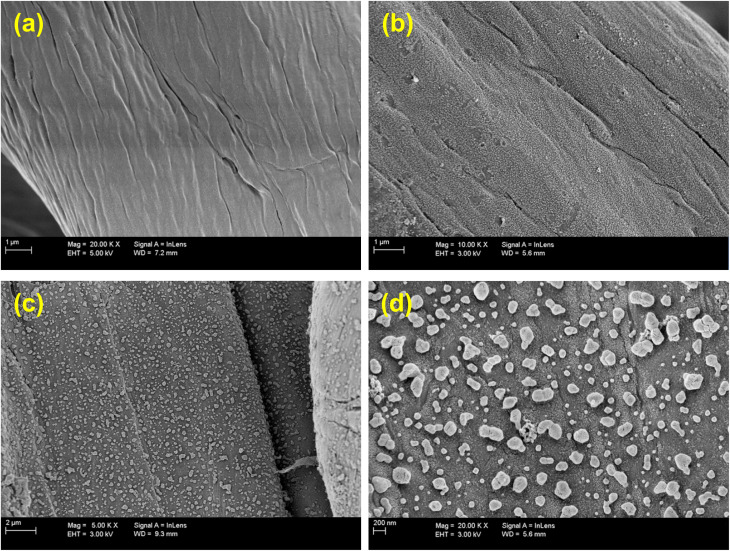
FE-SEM images of pristine CT (a), TiO_2_/CT (b), and Ag-Ag_2_S/TiO_2_/CT (c and d).

The morphologies of the prepared textile samples were studied *via* FE-SEM, as depicted in Fig. 3. In the images of the unmodified textile (Fig. 3a), details of the cellulose fibers forming the textile can be seen. After the textile was modified with TiO_2_ (Fig. 3b), these fibers were no longer as clear as before modification as a result of the appearance of a layer of TiO_2_ covering the surface of these fibers. For Ag-Ag_2_S/TiO_2_/CT (Fig. 3c), homogeneously distributed particles appeared on the textile fibers. At high magnification (Fig. 3d), particles that were expected to be Ag-Ag_2_S were observed scattered over the layer of TiO_2_.

Overall, the obtained results proved the successful functionalization of the textile with both Ag-Ag_2_S and TiO_2_, while retaining its properties and structure. Therefore, this functionalization method can be utilized with all substrates with low thermal resistance (*e.g.*, silk, plastic, paper, and wood), as conventional methods require a high calcination process for the synthesis of TiO_2_ anatase, which causes damage to the substrate and loss of its properties and structure. Besides, the fabrication process adopted in the current work is easy, simple, environmentally friendly, and inexpensive because it does not require a lot of chemicals and also does not consume a lot of energy (thermal and electrical).

### 3.4. XPS characterization

**Fig. 4:**
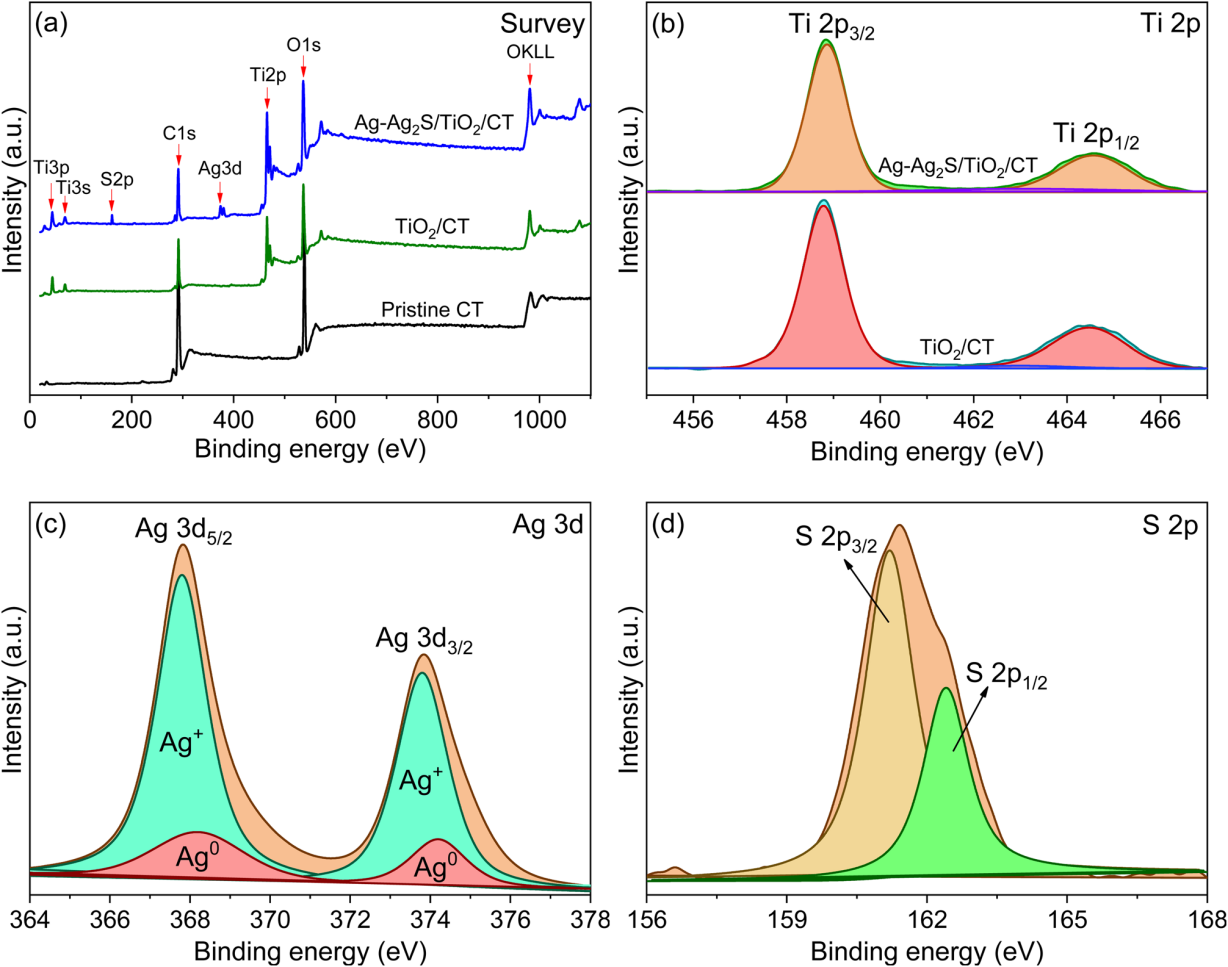
(a) XPS survey spectra of pristine CT, TiO_2_/CT, and Ag-Ag_2_S/TiO_2_/CT; (b–d) high-resolution XPS spectra: (b) Ti 2p, (c) Ag 3d, and (d) S 2p.

The chemical compositions of the textile samples were characterized *via* XPS. Fig. 4 displays the XPS spectra of the unmodified and modified textiles (TiO_2_/CT and Ag-Ag_2_S/TiO_2_/CT). Fig. 4a shows that the pristine textile consists of only the elements carbon and oxygen. After modifying the textile with TiO_2_, new peaks ascribed to titanium were observed. These peaks were found at 458.8 eV and 464.5 eV and were respectively attributed to Ti 2p_3/2_ and Ti 2p_1/2_ of TiO_2_,^61,62^ as presented in Fig. 4b. Their difference in binding energy was 5.8 eV, which means that the state of oxidation is Ti^4+^, which is related to the anatase TiO_2_.^61,62^

For Ag-Ag_2_S/TiO_2_/CT (Fig. 4a), new peaks attributed to sulfide and silver were discovered along with peaks observed in the TiO_2_ spectrum. The peaks of silver attributed to Ag 3d_5/2_ and Ag 3d_3/2_ can be dismantled into four peaks, as presented in Fig. 4c. The peaks at 368.2 eV and 374.2 eV are associated with Ag^0^,^20,25,63,64^ whereas the peaks at 367.8 eV and 373.8 eV are associated with Ag^+^ ions of Ag_2_S.^20,25,61,64–66^ The XPS spectrum of S 2p (Fig. 4d) presents two peaks at 161.2 eV and 162.4 eV for S 2p_3/2_ and S 2p_1/2_, respectively, which are related to S^2−^ ions in Ag_2_S.^20,35,65^ The binding energy difference between S 2p_1/2_ and S 2p_3/2_ is 1.2 eV, which corresponds to the values reported in the literature and indicates the production of Ag_2_S.^25,44^ Overall, the XPS results correspond to XRD and Raman results, which have proven the successful functionalization of textile fibers with Ag-Ag_2_S NPs and TiO_2_ anatase.

### 3.5. Optical properties

**Fig. 5:**
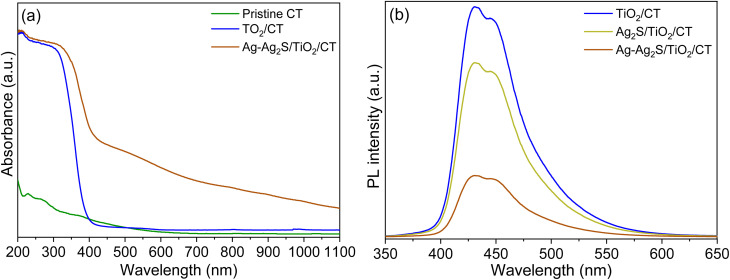
(a) UV-Vis-NIR absorption spectra of pristine CT, TiO_2_/CT, and Ag-Ag_2_S/TiO_2_/CT; (b) PL spectra of TiO_2_/CT, Ag_2_S/TiO_2_/CT, and Ag-Ag_2_S/TiO_2_/CT.

The band gap value of a photocatalyst plays an essential role in the activity of photocatalysis. Usually, the narrower the band gap of a photocatalyst, the greater its ability to absorb a large amount of visible light. Fig. 5a shows the absorbance spectra of the unmodified and modified textile (TiO_2_/CT and Ag-Ag_2_S/TiO_2_/CT). The unmodified textile was able to absorb only slightly in the UV region. After being modified with TiO_2_, its absorption in the ultraviolet domain improved significantly. However, it remained unable to absorb well in the visible region, with an absorption edge of about 390 nm. The Ag-Ag_2_S-modified cotton textile exhibited a light absorption band covering the ultraviolet, visible, and near-infrared regions (Fig. S1), with the appearance of a surface plasmon resonance (SPR) band at ∼400 nm, mainly attributed to Ag^0^, which is consistent with previous reports.^20,28,45,65,67^ TiO_2_/CT exhibited a wide visible light absorption range after coupling with Ag-Ag_2_S, which is consistent with previous reports.^25,28,53^ The good efficiency of Ag-Ag_2_S/TiO_2_/CT in the visible light absorption can be ascribed to the synergistic effect produced after the coupling of TiO_2_ with Ag-Ag_2_S as a plasmonic photocatalyst sensitive to visible light.^25,26,28,29,45,53,65^

The separation of photogenerated charges of textile samples was studied using PL. Separating the charges and preventing their recombination is essential in the photocatalytic process. A lower PL indicates a lower charge recombination. As shown in Fig. 5b, the TiO_2_/CT exhibited a high PL intensity around 430 nm, which significantly decreased after coupling with Ag_2_S NPs.^68,69^ This decrease indicates that the charge recombination process in TiO_2_ was inhibited, thanks to the improved separation and transfer of charges between TiO_2_ and Ag_2_S NPs.^68,69^ The PL peak of Ag_2_S/TiO_2_/CT was significantly higher than that of Ag-Ag_2_S/TiO_2_/CT, indicating that the photoinduced charge recombination rate of Ag_2_S/TiO_2_/CT was faster than that of Ag-Ag_2_S/TiO_2_/CT. This was due to the Ag NPs, which could minimize photoinduced charge recombination. This result also indicates the important role of Ag NPs in improving the photogenerated charge separation of Ag-Ag_2_S/TiO_2_/CT. Similar PL results were reported for Ag/AgCl/polydopamine-TiO_2_,^70^ Ag/AgBr/TiO_2_,^71^ and g-C_3_N_4_/Ag/TiO_2_ (ref. 72) systems. Suppressing the recombination of charges and improving their separation may enhance the interaction of these charges with H_2_O and O_2_, and thus improve the production of highly reactive oxygen species (ROS), which are primarily responsible for the degradation of organic molecules through the oxidation process.^27,68,69,73,74^

### 3.6. Photocatalytic degradation of *o*-toluidine in the liquid phase

*o*-Toluidine (TOD) is used in several industrial applications such as petroleum, dyes, rubber, and pharmaceuticals,^75,76^ which has significantly increased its discharge into the aquatic environment.^77^ The TOD causes several diseases, such as cancer of the urinary bladder and renal pelvis. It has been classified by the International Agency for Research on Cancer (IARC) as a dangerous substance that causes cancer.^78–80^ Therefore, it is important to treat water containing TOD using an efficient and safe technique.

**Fig. 6:**
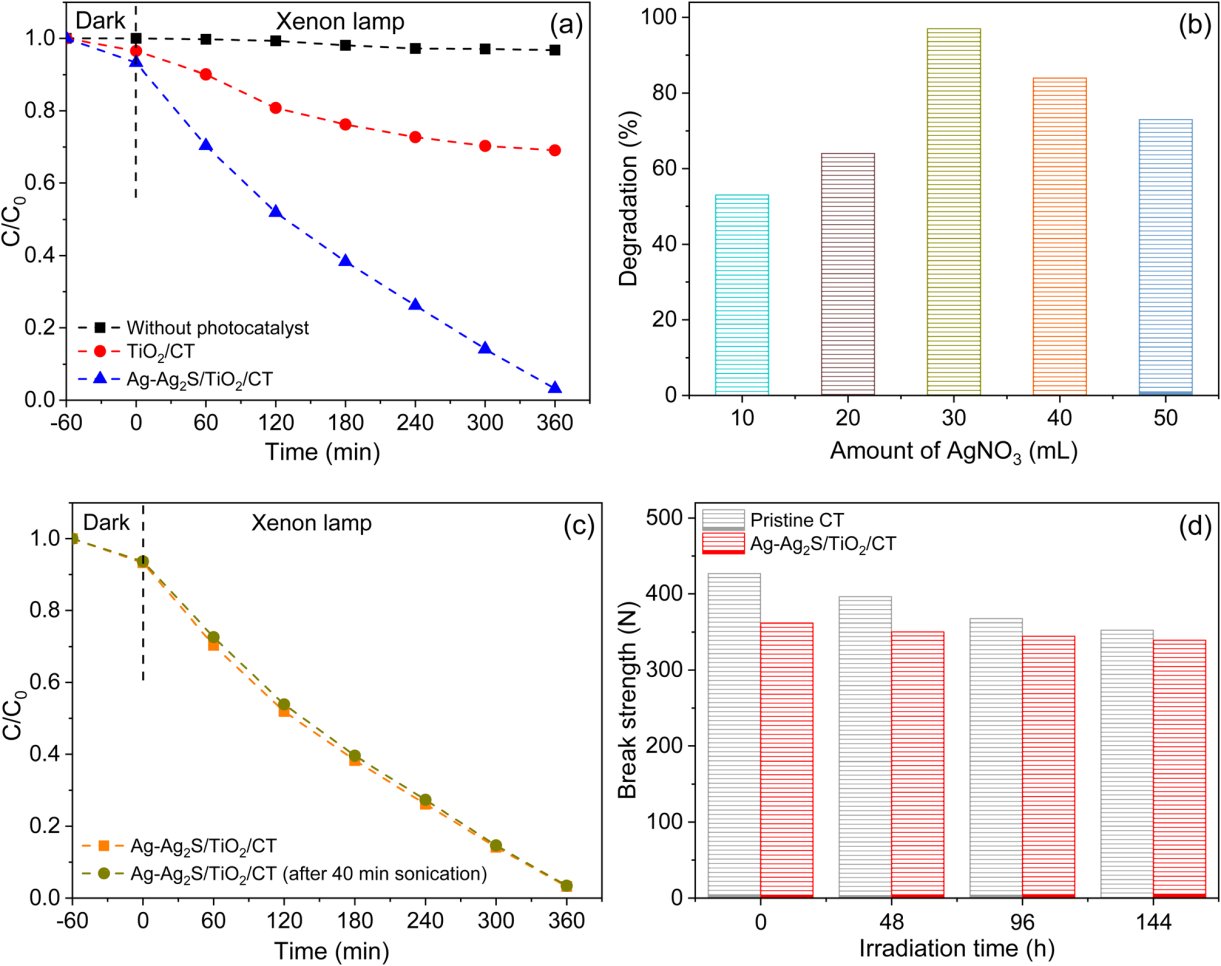
(a) TOD degradation curves; (b) the effect of the amount of AgNO_3_ solution as a precursor of Ag-Ag_2_S on the degradation of TOD; (c) evaluation of TOD concentration in the presence of Ag-Ag_2_S/TiO_2_/CT with and without sonication for 40 min; (d) break strength of the pristine textile and Ag-Ag_2_S/TiO_2_/CT with and without irradiation.

In this context, the efficacy of the textile samples in the water treatment for TOD as a model of carcinogenic compounds has been verified. The absorption rate of TOD by modified textile samples was negligible after 1 h under dark conditions. The concentration of TOD did not decrease when the unmodified textile was used under illumination, as presented in Fig. 6a. Modified textile samples showed improvement in the degradation of TOD, especially when TiO_2_/CT was associated with Ag-Ag_2_S NPs. The degradation rate of TOD after 6 h of illumination was 31% and 97% for both TiO_2_/CT and Ag-Ag_2_S/TiO_2_/CT, respectively. Table S1 displays the photocatalytic superiority of Ag-Ag_2_S/TiO_2_/CT in TOD degradation by comparing its activity with the photocatalytic activities of several reported photocatalysts.

The kinetic analysis of TOD degradation matched the pseudo-first-order kinetic model (Fig. S2). The kinetic constant (*k*) for Ag-Ag_2_S/TiO_2_/CT was ∼7 times greater than that of TiO_2_/CT, as shown in Table S2. This proves the crucial role played by Ag-Ag_2_S NPs after their association with TiO_2_ in the degradation of TOD molecules. The significant improvement in the efficacy of Ag-Ag_2_S/TiO_2_/CT in TOD degradation may be mainly attributed to the following: (i) the synergistic effect between Ag-Ag_2_S and TiO_2_, which includes the high ability to absorb visible light compared to TiO_2_/CT as shown in the DRS results (Fig. 5a); (ii) the inhibition of charge recombination due to improved separation as shown in PL results (Fig. 5b), and (iii) the smooth transfer of charges between Ag, Ag_2_S, and TiO_2_,^25,27–29^ which helps promote the generation of ROS.

To verify the existence of superoxide radical anions (˙O_2_^−^) and hydroxyl radicals (˙OH) in the photocatalytic reaction systems under illumination, electron spin resonance (ESR) experiments were performed in the presence of Ag-Ag_2_S/TiO_2_/CT. As shown in Fig. S3, DMPO−˙O^−^_2_ and DMPO−˙OH signals were detected in the presence of light; no signals were detected in the dark. Therefore, ˙O_2_^−^ and ˙OH radicals are considered the main active species that play an important role in photocatalysis.

To investigate the effect of the Ag-Ag_2_S quantity in the process of degradation of TOD, a series of photocatalytic experiments was carried out for Ag-Ag_2_S/TiO_2_/CT photocatalysts prepared with different amounts of AgNO_3_ solution (10–50 mL), as presented in Fig. 6b. The degradation rate of TOD of all Ag-Ag_2_S/TiO_2_/CT photocatalysts was better than that of TiO_2_/CT alone, proving the crucial role of Ag-Ag_2_S NPs in the degradation process of TOD. Furthermore, the rate of decomposition of TOD changed with the change in the amount of AgNO_3_ solution used. The best degradation rate of TOD was when using 30 mL of AgNO_3_, which was 97%. Therefore, 30 mL is considered the optimal amount for use in the preparation of the Ag-Ag_2_S/TiO_2_/CT photocatalyst.

The PL results of the Ag-Ag_2_S/TiO_2_/CT photocatalysts (Fig. S4) were identical to the results of the TOD degradation of those photocatalysts (Fig. 6b). The more the charge separation process is enhanced, the greater the degradation of TOD. This may indicate the effect of the amount of AgNO_3_ solution as a precursor to Ag-Ag_2_S in the charge separation process. The weakest PL intensity was when 30 mL of AgNO_3_ was used, the same amount detected when the best rate of TOD degradation was achieved. Generally, the amount of Ag-Ag_2_S loaded on the surface of TiO_2_/CT greatly affects the charge separation process and affects the performance of the photocatalyst, which is evident in the agreement between the PL results and the photocatalytic experiments of the Ag-Ag_2_S/TiO_2_/CT photocatalyst.

Stability, reusability, and durability are crucial features in the photocatalytic process, and a range of experiments were conducted to verify these properties. To check the adhesion strength of both Ag-Ag_2_S and TiO_2_ on the surface of the fabric fiber, Ag-Ag_2_S/TiO_2_/CT was treated with ultrasound for 40 min, and then its photocatalytic activity was checked. As presented in Fig. 6c, the activity of Ag-Ag_2_S/TiO_2_/CT did not change after sonication treatment and remained almost the same as before. There was no indication of disintegration or degeneration of Ag-Ag_2_S/TiO_2_/CT after sonication treatment. This proves the durability of Ag-Ag_2_S/TiO_2_/CT and the adhesion strength of both Ag-Ag_2_S and TiO_2_ on the surface of the textile fiber, allowing it to be easily recovered and reused as compared to photocatalysts in powder form, which require a filtration process in order to recover them from photocatalytic reactions.

In order to confirm the long-term stability of the Ag-Ag_2_S/TiO_2_/CT photocatalyst, it was reused for several consecutive cycles in the process of degradation of TOD under the same experimental conditions. As presented in Fig. S5, the rate of degradation of TOD did not change significantly after reuse, as the rate of degradation in the first cycle was 97%, while in the 20th cycle it was 85%. This proves the effectiveness of the Ag-Ag_2_S/TiO_2_/CT photocatalyst in the process of TOD degradation, its stability, and the possibility of its use for continuous cycles, which is important from an environmental and economic standpoint.

The lighting or modification process performed on the cotton textile (CT) using Ag-Ag_2_S and TiO_2_ can affect its mechanical properties. For this purpose, the tensile properties of the unmodified textile and the textile modified with Ag-Ag_2_S/TiO_2_ were verified (Fig. 6d). Before the lighting, the unmodified textile showed greater breaking strength compared with that of Ag-Ag_2_S/TiO_2_/CT (Fig. 6d), possibly due to the hydrothermal treatment performed on the textile to generate TiO_2_ on its fibers. After the lighting, the breaking strength of the unmodified textile was significantly decreased compared to Ag-Ag_2_S/TiO_2_/CT. After 144 h of lighting, the break strength retention ratios of the unmodified textile and Ag-Ag_2_S/TiO_2_/CT were 82.6 and 93.8%, respectively (Fig. S6), proving that the mechanical properties of the modified textile (Ag-Ag_2_S/TiO_2_/CT) were not remarkably affected by lighting as compared with the pristine textile. This may be attributed to the important role played by the Ag-Ag_2_S and TiO_2_ layer loaded on the textile in protecting the textile fiber from damage and the collapse of cellulose chains that may result from the photooxidation process.^81–83^

### 3.7. Photocatalytic degradation of alcohols in the gas phase

Gaseous alcohols, especially ethanol, 1-propanol, and 1-butanol, are used in several industrial applications, *e.g.*, fuels, paints, organic solvents, and plasticizers, as well as in the cosmetic and pharmaceutical industry. They can also be used in other fields such as medicine and agriculture, increasing their emissions as gases and thus air pollution.^84–89^ These gaseous compounds are characterized by their irritating odor and may cause some health problems, such as headaches, heart disease, dizziness, nervous system disorders, and respiratory disease.^84–89^ Therefore, it is necessary to purify the air from these gases using an effective, reliable, and safe technology.

**Fig. 7:**
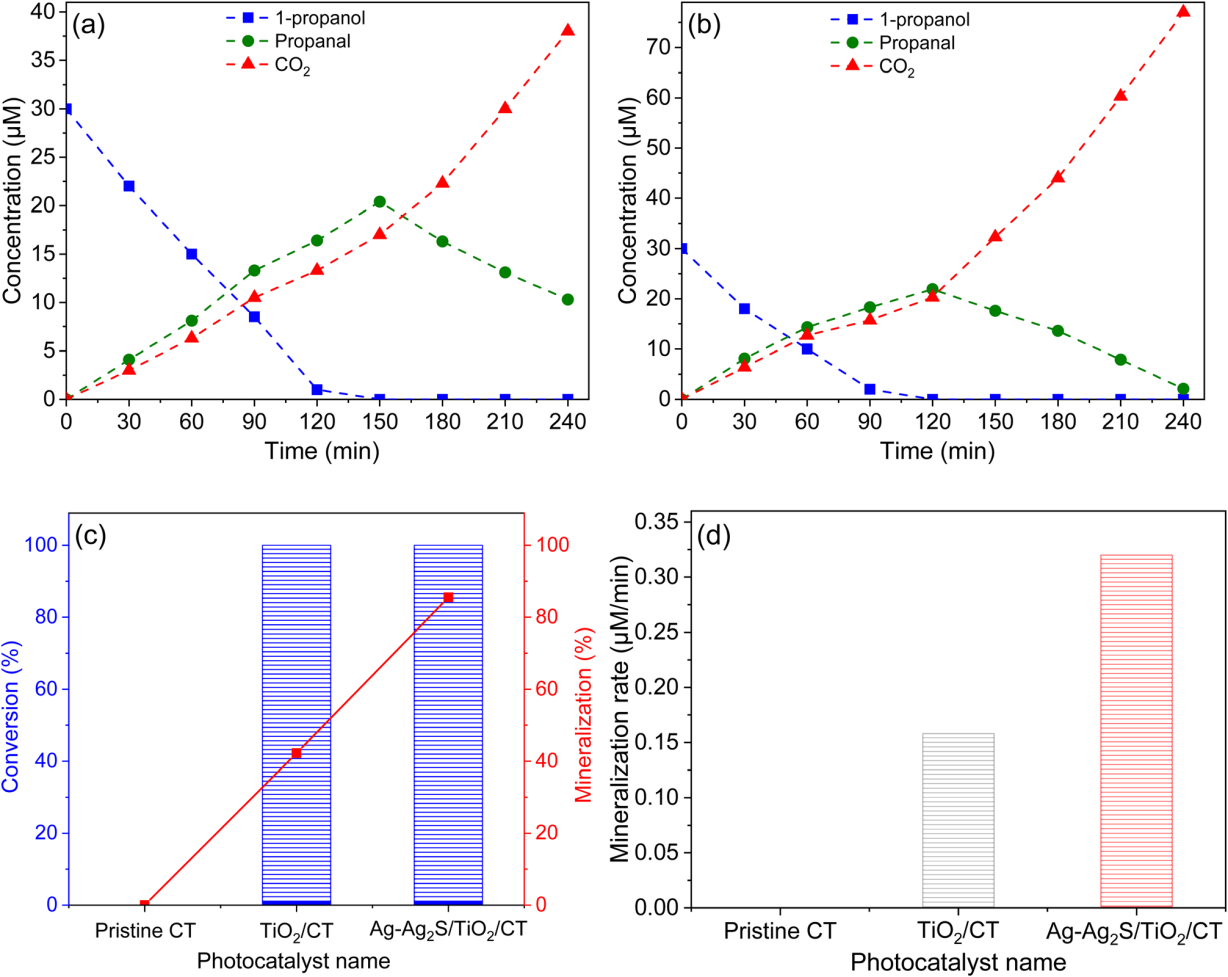
Photodegradation curves of 1-propanol and the production of CO_2_ and propanal *vs.* irradiation time *via* TiO_2_/CT (a) and Ag-Ag_2_S/TiO_2_/CT (b). The mineralization and conversion ratio of 1-propanol (c), and the mineralization rate of 1-propanol (d).

In this context, the efficacy of the textile samples in air purification from gaseous alcohols using 1-propanol as a model of VOCs has been verified (Fig. 7). Under dark conditions, the adsorption ability of all photocatalysts was negligible. Besides, the blank experiments displayed no decrease in the concentration of 1-propanol, which indicates the importance of light, catalyst, and oxygen in the oxidation process of 1-propanol. The concentration of 1-propanol decreased in the presence of modified textiles under lighting (Fig. 7). The decrease in concentration of 1-propanol during the photocatalytic process coincided with the production of propanal as an intermediate product and CO_2_ as the final product. Interestingly, the concentration of propanal decreased after the complete decomposition of 1-propanol, while the concentration of CO_2_ continued to rise, which may indicate that the 1-propanol was converted into propanal first, and then to CO_2_. The decrease in 1-propanol concentration and the production of CO_2_ were faster in the presence of Ag-Ag_2_S/TiO_2_/CT (Fig. 7b) compared to TiO_2_/CT (Fig. 7a), where the mineralization rate of 1-propanol was 42.2% and 85.5% after 4 h of lighting for TiO_2_/CT and Ag-Ag_2_S/TiO_2_/CT, respectively, despite the degradation rate of 1-propanol being 100% for both photocatalysts after 4 h of lighting (Fig. 7c). The initial mineralization ratio (*r* = d[CO_2_]/d*t*) (Fig. 7d) was remarkably influenced by the kind of hybrid photocatalyst where the superiority of Ag-Ag_2_S/TiO_2_/CT over TiO_2_/CT was evident. The photocatalytic superiority of Ag-Ag_2_S/TiO_2_/CT compared to TiO_2_/CT can be ascribed mainly to the plasmon effect of Ag-Ag_2_S in inhibiting charge recombination and improving visible light absorption, which contributed to the formation of more ROS responsible for oxidizing 1-propanol.

In order to verify the efficacy of Ag-Ag_2_S/TiO_2_/CT in purifying air from VOCs, specifically alcoholic gases, photocatalytic degradation tests were performed on ethanol (Fig. S7a) and 1-butanol (Fig. S7b). Under lighting, the concentration of target gases decreased over time along with the production of intermediate products and CO_2_. The main intermediate products of 1-butanol and ethanol were butanal and ethanal, respectively. The concentrations of these intermediate products decreased after the complete decomposition of the target gases, whereas the CO_2_ concentration continued to rise. The decrease in ethanol concentration (Fig. S7a) was faster compared to the concentrations of other target gases (1-propanol (Fig. 7b) and 1-butanol (Fig. S7b)). The complete decomposition of ethanol occurred after ∼60 min, while the complete degradation of 1-butanol and 1-propanol occurred after ∼120 and ∼90 min, respectively. Interestingly, the concentrations of intermediate products increased during the decomposition of their original gas, with each intermediate product reaching its peak concentration with the completion of the decomposition of its original gas. This result indicates that the decomposition of alcoholic gases is closely related to the production of its intermediate aldehyde. After 4 h of light, the mineralization ratios of 1-butanol (C_*n*_H_2*n*+1_OH (*n* = 4)) and ethanol (C_*n*_H_2*n*+1_OH (*n* = 2)) were 60.8% and 95%, respectively, compared to 85.5% for 1-propanol (C_*n*_H_2*n*+1_OH (*n* = 3)) (Fig. S7c), although the decomposition rate of all target gases was 100%. This result indicates that the mineralization rate of alcoholic gases is related to the number of carbon atoms in their structure, where, when these atoms increase, the rate of mineralization decreases. Table S3 displays the photocatalytic superiority of Ag-Ag_2_S/TiO_2_/CT in the degradation of alcoholic gases by comparing its activity with the photocatalytic activities of several reported photocatalysts.

The stability of the photocatalyst is an important factor in practical applications. Therefore, Ag-Ag_2_S/TiO_2_/CT was reused for three cycles under similar experimental conditions to verify its efficiency and stability. As presented in Fig. S7d, the mineralization rates of alcoholic compounds did not change remarkably after the three cycles, proving the efficacy and stability of Ag-Ag_2_S/TiO_2_/CT toward alcoholic gases.

### 3.8. Antimicrobial properties

**Fig. 8:**
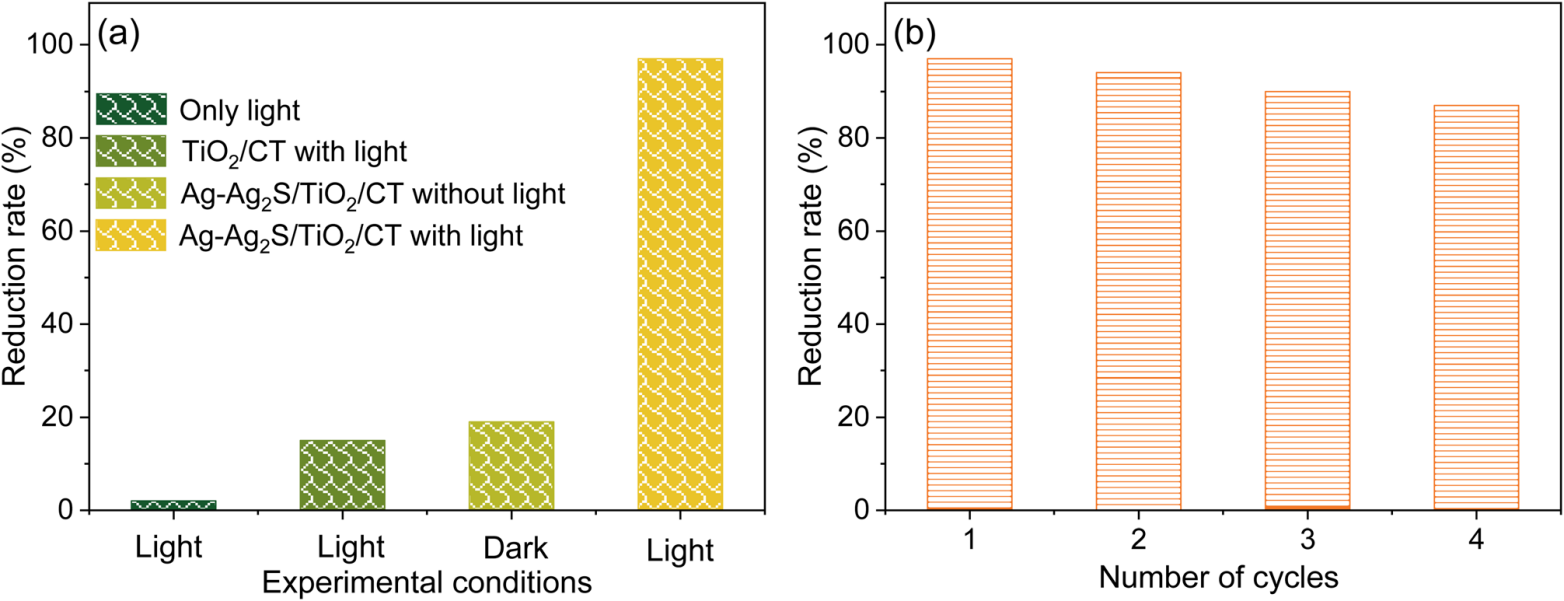
(a) Photocatalytic disinfection of *E. coli* with and without light for 1 h; (b) the reusability of Ag-Ag_2_S/TiO_2_/CT for the inactivation of *E. coli*.

In order to verify the antimicrobial properties of the textile samples, experiments were conducted to disinfect water from *E. coli* as a pathogenic bacterium (Fig. 8a). In the case of unmodified tissue under light conditions, no disinfection of *E. coli* was detected. In contrast, tissue modified with TiO_2_ showed weak antibacterial activity after 1 h of illumination. This activity improved significantly after the coupling of TiO_2_/CT with Ag-Ag_2_S, with the bacteria reduction rate of Ag-Ag_2_S/TiO_2_/CT reaching 97% after 1 h under light. However, when the experiment was carried out in dark conditions, the bacterial reduction rate of Ag-Ag_2_S/TiO_2_/CT decreased to 19% after 1 h. It is assumed that the high antibacterial activity that occurred under the light is due to the photocatalytic process, where ROS can be generated by the photoexcitation of Ag-Ag_2_S/TiO_2_/CT with xenon light. ROS can kill bacteria by damaging the cell envelope and cell components (ribosomes, proteins, DNA, peptidoglycan layer, *etc.*).^39,52,90,91^ The antibacterial effect that occurred in dark conditions can be produced by both Ag and Ag_2_S, where Ag NPs can cause cell damage to bacteria by attaching to the surface of the membrane or penetrating inside the cell,^39,92^ and Ag_2_S can release Ag^+^ ions, which kill bacterial cells by disrupting the enzyme and disintegrating the cell wall.^39,92,93^ These results demonstrate that the active species produced *via* the photocatalytic process are key to inactivating bacteria under light, and indicate the important role that Ag-Ag_2_S plays in enhancing the efficiency of TiO_2_/CT in the disinfection process. Table S4 highlights the high efficacy of Ag-Ag_2_S/TiO_2_/CT in inactivating *E. coli* bacteria by comparing its efficiency with several other reported photocatalysts.

The reusability of the photocatalyst and the stability of its performance for long times are important in terms of sustainability and practicality. For this purpose, the reusability of Ag-Ag_2_S/TiO_2_/CT was investigated for four cycles to prove its stability (Fig. 8b). The reduction rate of *E. coli* was not significantly changed after four cycles, reaching 97% in the first cycle, while in the fourth cycle it reached 87%, demonstrating the reusability of Ag-Ag_2_S/TiO_2_/CT and its stable effectiveness against pathogenic bacteria (*E. coli*). These results indicate that Ag-Ag_2_S/TiO_2_/CT is a promising antimicrobial agent that can be used under sunlight to disinfect water contaminated with bacteria.

### 3.9. The leakage of silver from Ag-Ag_2_S/TiO_2_/CT

According to international standards, the concentration of Ag should not exceed 0.1 ppm in drinking water;^94^ if this level is exceeded, poisoning and environmental hazards may occur. In addition, silver leakage from Ag-based catalysts may cause the low efficiency and instability of the catalysts; therefore, the amount of silver leaking from Ag-based materials must be determined. For this purpose, Ag-Ag_2_S/TiO_2_/CT (4 cm × 4 cm) was placed in 50 mL of distilled water under light conditions for 24 h, and the amount of Ag was determined *via* ICP. The amount of Ag leached was 0.03 ppm after 24 h, respectively, which is consistent with international standards. This result proves that Ag-Ag_2_S/TiO_2_/CT is a stable photocatalyst and does not represent a risk to aquatic ecosystems, allowing it to be used as an antibacterial material in aquatic media.

### 3.10. Possible photocatalytic mechanism

**Scheme 1:**
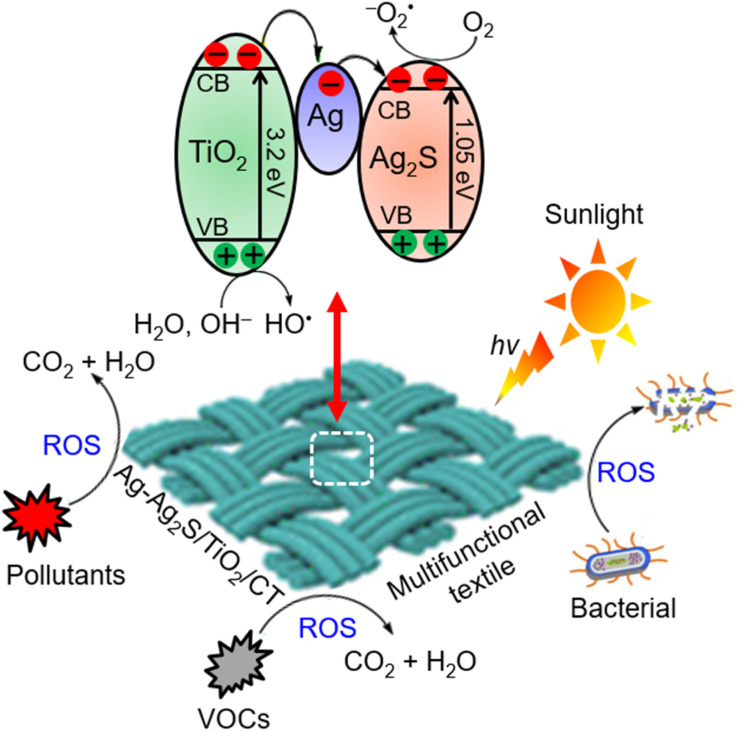
Proposed photocatalytic mechanism of Ag-Ag_2_S/TiO_2_/CT under sunlight.

The charge mobility pathway and photocatalytic mechanism of Ag-Ag_2_S/TiO_2_/CT under sunlight can be proposed based on experimental results and literature data.^25,29^ The valence band (VB) and conduction band (CB) edge potentials of Ag_2_S with a band gap of 1.05 eV are located at 0.995 eV and −0.055 eV (*vs.* NHE), respectively,^41^ while the VB and CB potentials of TiO_2_ with a band gap of 3.18 eV are located at 2.86 eV and −0.32 eV (*vs.* NHE), respectively.^41^ Under sunlight, both TiO_2_ and Ag_2_S can be excited because sunlight contains ∼5% UV light and ∼43% visible light.^95–97^ The photocatalytic mechanism of Ag-Ag_2_S/TiO_2_/CT is based on the type II heterojunction with the role of Ag as a solid mediator for electron capture and transfer, as presented in Scheme 1.^25,29^ The photogenerated electrons can be transferred from the CB of TiO_2_ to the metal Ag and then the CB of Ag_2_S, due to the CB potentials of both Ag_2_S and TiO_2_ and the Fermi levels of metallic Ag located at −4.50 eV, −4.21 eV, and −4.26 eV from the vacuum energy level, respectively.^20,25,29,41,63^ This smooth electron transfer between Ag, Ag_2_S, and TiO_2_ helps inhibit recombination.^25,29^ The electrons in the CB of Ag_2_S can react with dissolved oxygen (O_2_) to produce superoxide radical anions (˙O_2_^−^), while the holes in the VB of TiO_2_ can react with hydroxide (OH^−^) and H_2_O to produce hydroxyl radicals (˙OH). These radicals can oxidize organic pollutants into CO_2_ and H_2_O and kill bacteria.

## 4. Conclusion

Herein, we have presented an easy approach to the synthesis of Ag-Ag_2_S/TiO_2_/CT as a highly efficient biocomposite for environmental and biological applications. The Ag-Ag_2_S/TiO_2_/CT biocomposite was synthesized *via* two steps involving a moderate hydrothermal process to produce TiO_2_/CT, followed by a dipping process in an Ag-Ag_2_S solution. The crystal structure properties of the biocomposite were verified *via* XRD and Raman measurements that proved the existence of anatase TiO_2_ and Ag-Ag_2_S on the textile surface. SEM images showed that a layer of TiO_2_ decorated with Ag-Ag_2_S NPs covering the textile fibers is formed. DRS and PL measurements showed improved optical properties of TiO_2_/CT after being coupled with Ag-Ag_2_S NPs. This biocomposite has shown a high decomposition efficiency of 97% toward TOD, a carcinogenic compound, in aqueous media after 6 h of lighting. It also showed complete oxidation of alcoholic gases (which include 1-butanol, 1-propanol, and ethanol) as VOCs under sunlight at room temperature. Moreover, it demonstrates notable antibacterial activity against *E. coli*. This high efficiency of Ag-Ag_2_S/TiO_2_/CT can be ascribed, in particular, to the synergistic effect between Ag-Ag_2_S and TiO_2_, which includes the smooth transfer of charges, thanks to the efficient separation of charge, recombination inhibition, and improved absorption of visible light. Experiments regarding tensile strength, durability, and reusability confirmed the stability of the hybrid photocatalyst, with the possibility of using it for a long time and the ease of its recovery. This work is the first based on TiO_2_-coated textiles decorated with Ag-Ag_2_S NPs as multifunctional biocomposites. To address environmental and biological pollution, this work can serve as a primary platform in the synthesis and design of textiles as multifunctional biocomposites that are activated under sunlight.

## Conflicts of interest

There are no conflicts to declare.

## Supplementary Material

RA-015-D5RA04993H-s001

## Data Availability

The authors confirm that all data have been included in the manuscript. Supplementary information is available. See DOI: https://doi.org/10.1039/d5ra04993h.
