# Supplementary material for: Simple and green fabrication of an Ag-Ag2S/TiO2/cellulose biocomposite film with enhanced photocatalytic and antibacterial activity
Source: RSC Adv. 2025 Sep 19;15(41):34225–36. doi: 10.1039/d5ra04993h (PMC12447267; doi:10.1039/d5ra04993h)
Supplement: RA-015-D5RA04993H-s001 [file RA-015-D5RA04993H-s001.pdf]

Supplementary Information (SI) for RSC Advances.
This journal is © The Royal Society of Chemistry 2025

Supporting Information

Simple and green fabrication of Ag-Ag_2S/TiO_2/cellulose biocomposite film with enhanced photocatalytic and antibacterial activity

Mouheb Sboui [a]*, Khalid A. Alamry [b], Youssef O. Al-Ghamdi [c], Balachandran Subramanian [d], Meenakshisundaram Swaminathan [e], Mahmoud A. Hussein [b,f]*

[a] Department of Chemistry, Faculty of Sciences, University of Sfax, Sfax BP1171-3018, Tunisia

[b] Chemistry Department, Faculty of Science, King Abdulaziz University, P.O. Box 80203, Jeddah 21589, Saudi Arabia

[c] Department of Chemistry, College of Science Al-Zulfi, Majmaah University, Al-Majmaah 11952, Saudi Arabia

[d] Functional Materials and Materials Chemistry Laboratory, Saveetha Dental College and Hospitals, Saveetha Institute of Medical and Technical Sciences, Saveetha University, Chennai 600 077, Tamil Nadu, India

[e] Nanomaterials Laboratory, Department of Chemistry, International Research Centre, Kalasalingam Academy of Research and Education, Krishnankoil 626126, India

[f] Chemistry Department, Faculty of Science, Assiut University, Assiut, 71516, Egypt

* Corresponding authors.

E-mail address: sboui.mouheb@gmail.com (M. Sboui); mahussein74@yahoo.com maabdo@kau.edu.sa, (M.A. Hussein).

S1. Synthesis of Ag-Ag_2S/TiO_2/CT biocomposite films

All the chemical reagents were purchased from Sigma-Aldrich and used as received. TiO_2/CT was prepared according to the following steps: The cotton textile (CT) was soaked in solution containing of 1.7 (w/v)% of tetra-butyl titanate and a mixture of tert–butanol plus acetic acid (90/10 *wt*%) for 8 h at room temperature. Then the CT was dried at 60 °C for 2 h before subjected to the hydrothermal treatment at 130 °C for 4 h. The sample was recovered, washed with water and dried again at 50 °C for 2 h.

For Ag-Ag_2S/TiO_2/CT, an aqueous solution of Ag_2S was prepared first at room temperature. Typically, $AgNO_3$ solution (30 mL, 5×10^{-3} M) was mixed with Na_2S solution (20 mL, 5×10^{-3} M) under continuous stirring in the dark for 3 h to generate Ag_2S. Subsequently, the formed Ag_2S suspension solution was exposed to Xenon lamp irradiation for 1 h under mild stirring to create Ag-Ag_2S. Finally, the TiO_2/CT sample was dipped in the prepared Ag-Ag_2S aqueous solution for 6 h. After taking the sample out of the Ag-Ag_2S solution, it was rinsed with deionized water to remove excess precursors and particles not attached to the cotton surface and then dried for 2 h at 50 °C. A series of photocatalysts was prepared using this procedure and changing the the amount of $AgNO_3$ solution between 10 mL and 50 mL.

For comparison, Ag_2S/TiO_2/CT sample was prepared using the same method as above, without exposing the resulting Ag_2S suspension to Xenon lamp irradiation.

S2. Characterization of photoactive textiles

The crystallographic properties of prepared materials were investigated on a Bruker AXS diffractometer (Bruker AXS, Madison, WI) with a Cu Kα radiation, generated at 30 kV and a

current of 100 mA, incident on small pieces of the cotton textile. The (2θ) angular region from 10° to 60° was scanned using steps of 0.05° with a step time of 10 s.

Raman spectra were recorded on a LabRAM Analytical Raman micro-spectrograph (Jobin-Yvon, Horiba group, Longjumeau Cedex, France) using anargon laser source as exciting radiation (λ = 532 nm) and an air-cooled CCD (Charge-Coupled Device) detector. The acquisition time was 100 s.

The surface morphologies of textile fibers were observed on a field emission scanning electron microscope (FE-SEM, ZEISS SUPRA40, UK). The electron source, a hot cathode producing electrons by Schottky effect, is a tungsten filament coated with a ZrO layer. The samples were attached on a carbon adhesive and coated with a thin layer of Pt with a thickness of about 1 nm by sputtering under an inert gas.

The surface chemical compositions of textile fibers were examined on an X-ray photoelectron spectroscope (XPS, PHI-5000C, PerkinElmer Inc., USA) with the C1s spectrum at 285.1 eV as the standard value. XPS data were collected using a 1486.6 eV Al Kα line. The power was 100 W, and the vacuum was smaller than 10^{-8} Torr.

The diffuse reflectance spectra (DRS) of prepared photoactive textiles in the 200–1100 nm wavebands were recorded at room temperature on the Shimadzu UV-3101PC UV-VIS-NIR Spectrophotometer (Shimadzu Corporation, Japan) equipped with an integrating sphere (θ = 60 mm) at a scanning speed of 200 nm/min. $BaSO_4$ powder was used as a reference.

The photoluminescent (PL) spectra of textile fibers were obtained on a Fluorescence Spectrometer (HORIBA Jobin Yvon Fluorolog-3) equipped with a Xenon lamp (100 W) at room temperature.

To investigate the presence of superoxide radical anions ($^{\bullet}O_2^-$) and hydroxyl radical ($^{\bullet}OH$) in the photocatalytic reaction systems under illumination (Xenon lamp (100 W)), electron spin resonance (ESR) experiments were performed in the presence of Ag-Ag_2S/TiO_2/CT using a Bruker model ER200-SRC spectrometer.

Testing of the mechanical properties of the prepared materials was performed on an Instron 3367 Universal Material Testing Machine (Instron Ltd., USA) according to standard PN-EN ISO 13934-1:2013-07.[1] Each sample was measured at least 10 times in different locations of the corresponding fabric, and the average value of these tests was reported.

The amount of Ag released from Ag-Ag_2S/TiO_2/CT was determined through inductively coupled plasma atomic emission spectrometer (ICP-AES Perkin-Elmer OPTIMA 3000). Mechanical properties testing of samples were performed on an Instron 3367 machine (Instron Ltd., USA).

S3. Photocatalytic experiments

S3.1. Photocatalytic experiments in liquid phase

The photocatalytic activity of prepared samples was investigated by the degradation of *o*-toluidine (TOD) under simulated-sunlight irradiation (Xenon lamp) at ambient condition. The photocatalytic test was carried out through the following path: a textile sample (6 cm × 6 cm) was placed in a crystallizer containing 50 mL of TOD aqueous solution (C_0 = 25 mg/L). Before irradiation, the mixture (photocatalyst + TOD solution) was kept in a dark facility for 1 h to establish adsorption equilibrium between the prepared sample and the contaminant (TOD). After that, the system was irradiated by a 100 W Xenon lamp placed at 10 cm from the top of the

crystallizer. During a specified period of illumination, the concentration of TOD was analyzed via a UV-Vis spectrophotometer (UV-6300, Mapada).

The degradation rate of TOD was calculated on the basis of equation (Eq. (S1)), and the first-order kinetics was also used to calculate a constant rate (k, min^{-1}) as shown in equation (Eq. (S2)):

$$\text{Degradation rate (\%)} = \frac{C_0 - C_t}{C_0} \times 100 \qquad \text{(S1)}$$

$$\ln\left(\frac{C_0}{C_t}\right) = k \times t \qquad \text{(S2)}$$

where C_0 (mg/L) is the initial concentration of TOD, and C_t (mg/L) is the concentration of TOD after a specified irradiation time (t).

To check the repeatability of the photocatalytic activity, the Ag-Ag_2S/TiO_2/CT was used in twenty consecutive photodegradation cycles of TOD under simulated-sunlight irradiation (Xenon lamp). After each run the photocatalyst was rinsed with water, dried and used again in the same conditions.

S3.2. Photocatalytic experiments in gas phase

To verify the efficiency and ability of the photocatalysts that were prepared in this study to remove 1-propanol ($C_nH_{2n+1}OH$ (n = 3)) in gas phase, a series of photodegradation experiments of 1-propanol was conducted in a cylindrical batch-type reactor of Pyrex glass (height: 14.0 cm,

diameter: 9.0 cm and volume of 890 cm^3). The photocatalytic textile was placed in the reactor as a 9 cm diameter circle.

Before starting the experiments, the reactor was purged of any gas by cleaning it with oxygen for 1 h. Thereafter, 1-propanol was injected by a septum located inside wall of the photoreactor. The initial concentration of 1-propanol in gas phase was stabilized at 30 µM.

The reactor was placed in the center of the SOLARBOX apparatus which has a Xenon lamp (500 W) simulating solar energy and placed at the top. It also contains a water filter located between the reactor and the lamp to keep the heat of reactor at room temperature. Before starting the irradiation process with the Xenon lamp, the apparatus was maintained in the dark for 1 h in order to balance the adsorption between the catalyst and the pollutant (1-propanol). The duration of one test for each catalyst took 4 h of illumination and 200 µL of gas was periodically taken from the reactor via a gas-tight syringe for analysis. GC-17A Shimadzu gas chromatograph with a HP-1 column and a flame ionization detector (FID) was used to monitor the concentration of 1-propanol and the intermediate products, whereas CO_2 content was quantified by an HP6890 gas chromatograph with a 60/80 Carboxen column and a TCD.

The percentage of 1-propanol conversion in the reactor is expressed by the following equation (Eq. (S3)):

$$\text{Conversion (\%)} = \frac{[\text{1-propanol}]_{in} - [\text{1-propanol}]_{out}}{[\text{1-propanol}]_{in}} \times 100 \qquad \text{(S3)}$$

where $[\text{1-propanol}]_{in}$ and $[\text{1-propanol}]_{out}$ are the molar concentrations at the inlet and outlet of 1-propanol, respectively.

The percentage of conversion to CO_2 was calculated using the following equation (Eq. (S4)):

$$\text{Mineralization (\%)} = \frac{[\mathrm{CO_2}]_{out}}{n \times [\text{1-propanol}]_{\text{in}}} \times 100 \qquad \text{(S4)}$$

where $[CO_2]_{out}$ represents the concentration of CO_2 measured at the outlet of the photocatalytic reactor and *n* represents the number of carbon atoms that 1-propanol contains.

To check the effectiveness of the prepared photocatalyst (Ag-Ag_2S/TiO_2/CT) in air purification, both ethanol ($C_nH_{2n+1}OH$ (n = 2)) and 1-butanol ($C_nH_{2n+1}OH$ (n = 4)) were used as models of VOCs. All the photocatalytic experiments were carried out under conditions similar to the 1-propanol test.

In addition, the photocatalytic stability of Ag-Ag_2S/TiO_2/CT was analyzed by reusability tests under the same experimental conditions. For that, three photocatalytic cycles were carried out, with an intermediate process between them that consisted of keeping the photocatalyst in the reactor under illumination and oxygen flow for 45 min in order to reactivate the photocatalyst and use it in the next cycle. The duration of each photocatalytic cycle is 4 h.

S3.3. Antibacterial experiments

S3.3.1. Bacteria preparation

The bacterial strains used as test organism in this study were *Escherichia coli* (*E. coli*) ATCC 25922. It should be noted that glassware used and the culture medium solution were autoclaved under 120 °C for 20 min to ensure sterility. All relevant experiments were performed under sterile environment. Frozen stock cultures (−80 °C) of bacteria were sub-cultured into nutrient agar and, then, transferred to a nutrient broth. The bacterial cells were cultivated on

Luria-Bertani (LB) broth as growth media for 18 h in a thermostatic incubator at 37 °C and culture was vigorously stirred to ensure aeration (150 rpm).

S3.3.2. Photocatalytic inactivation

The photocatalytic disinfection of the prepared samples was carried out in dark and light conditions using a 100 W Xenon lamp as the light source. All glass apparatuses used in the experiments and the textile samples used as photocatalysts were washed with deionized water, and then autoclaved at 120 °C for 20 min to ensure sterility. 10 mL of bacterial cells were collected from an overnight culture in Luria-Bertani (LB) agar plates, harvested by centrifugation (10,000 rpm for 3 min), washed three times with a proper sterilized physiological saline solution (0.9% of NaCl at pH = 7.0) to remove the residual growth medium and put in 10 mL of distilled water (bacterial stock solution). Experiments were carried out as follows: a textile sample (4 cm × 4 cm) was soaked in 50 mL in bacteria suspension of 1×10^7cfu mL^{-1} and the glass flask was shaken with an orbital shaker. The system was then illuminated by a 100 W xenon lamp placed at 20 cm from the top of the beaker. Aliquot samples were collected at different time intervals and diluted with sterilized saline solution, then immediately spread on LB agar plates and incubated at 37 °C for 24 h to determine the number of viable cells (in cfu). For comparison, experiments were also conducted under darkness or in the presence of untreated textile. All the treatment and control experiments were performed in triplicate.

The bacterial reduction rate of each test was calculated using equation (Eq. (S5));

$$\text{Reduction rate (\%)} = \frac{N_0 - N_1}{N_1} \times 100 \qquad \text{(S5)}$$

where N_0 and N_1 are the initial number of viable cells and the number of viable cells after irradiation, respectively.

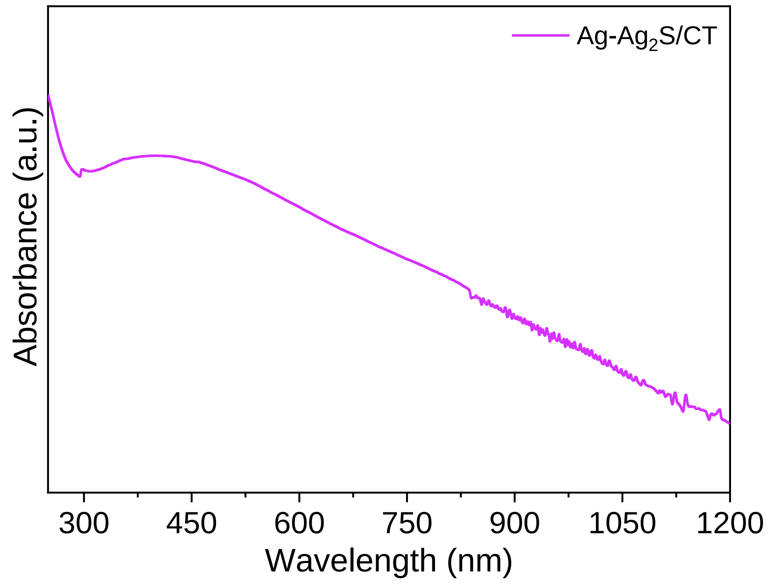

Fig. S1. UV–Vis–NIR absorption spectrum of Ag-Ag_2S/CT.

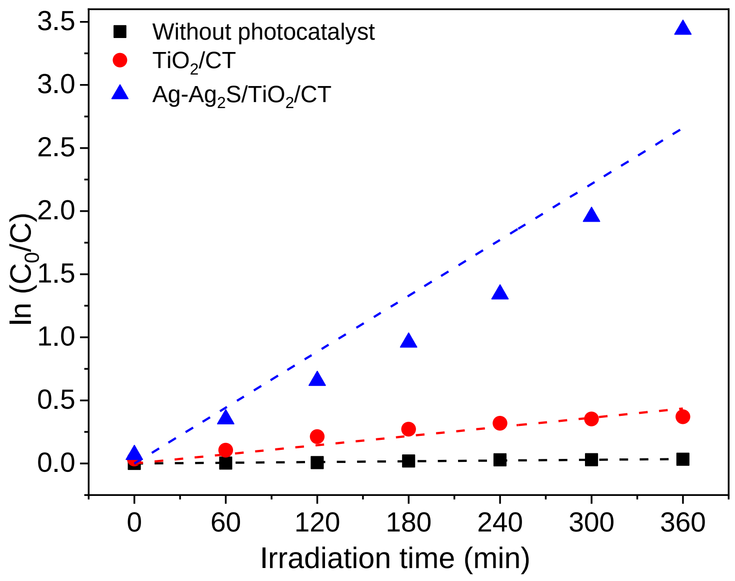

Fig. S2. Pseudo-first-order kinetics of TOD.

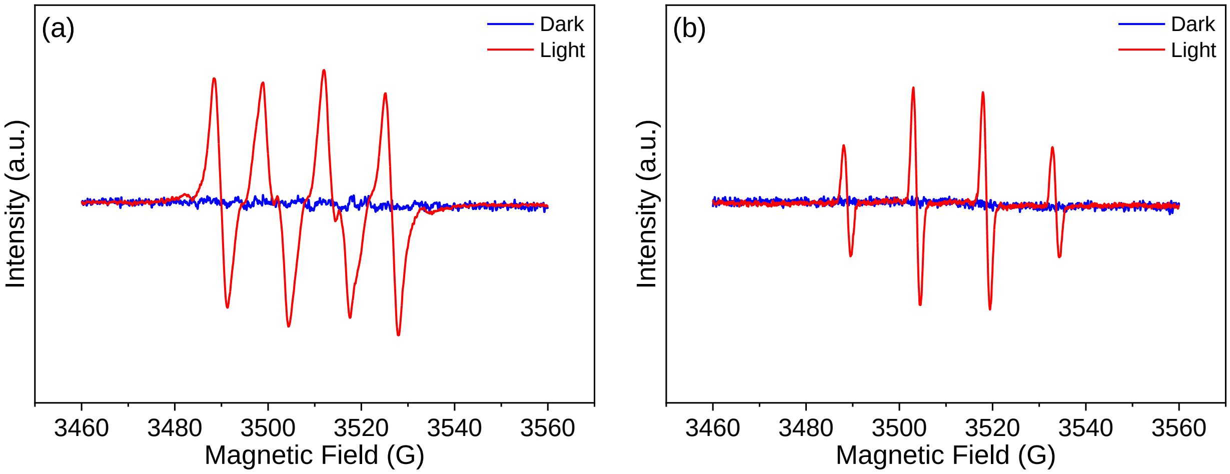

Fig. S3. The ESR spectra of DMPO−$^{\bullet}O^{-}_{2}$ (a) and DMPO−$^{\bullet}$OH (b) in the presence of the Ag-Ag_2S/TiO_2/CT sample.

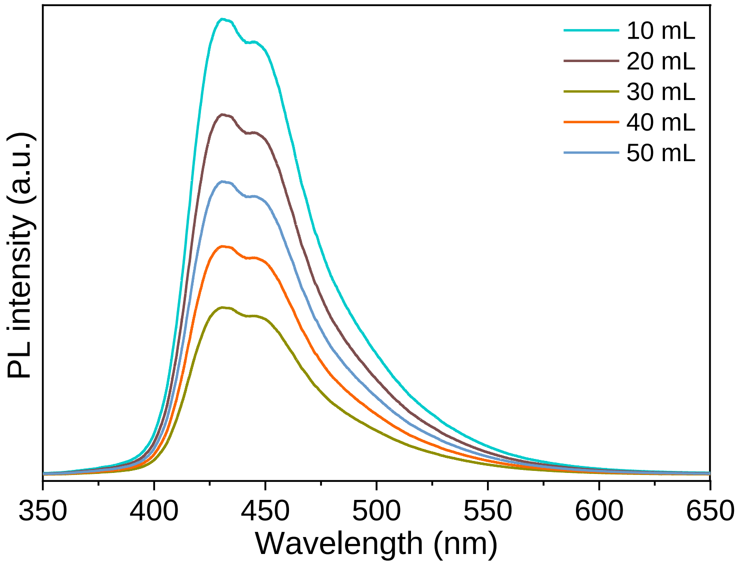

Fig. S4. PL spectra of Ag-Ag_2S/TiO_2/CT prepared with different amount of $AgNO_3$ solution as a precursor of Ag-Ag_2S/TiO_2.

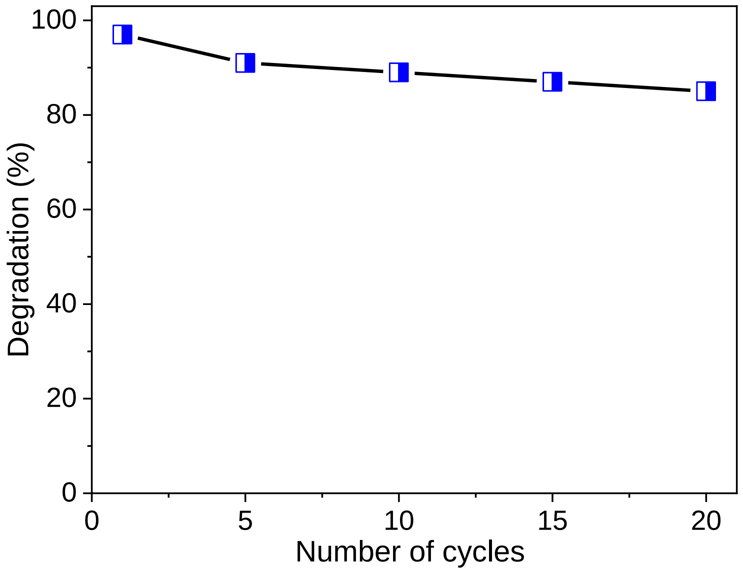

Fig. S5. Reusability of Ag-Ag_2S/TiO_2/CT for photodegradation of TOD during successive cycles.

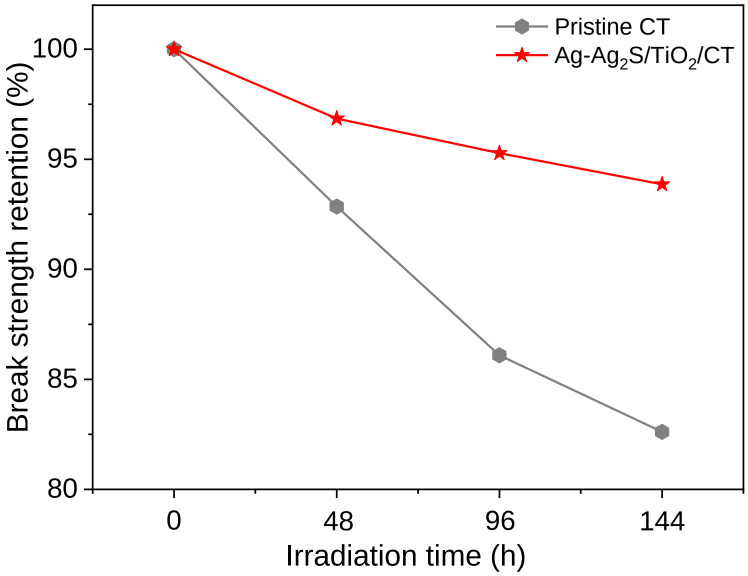

Fig. S6. Break strength retention of the untreated textile and Ag-Ag_2S/TiO_2/CT before and after irradiation.

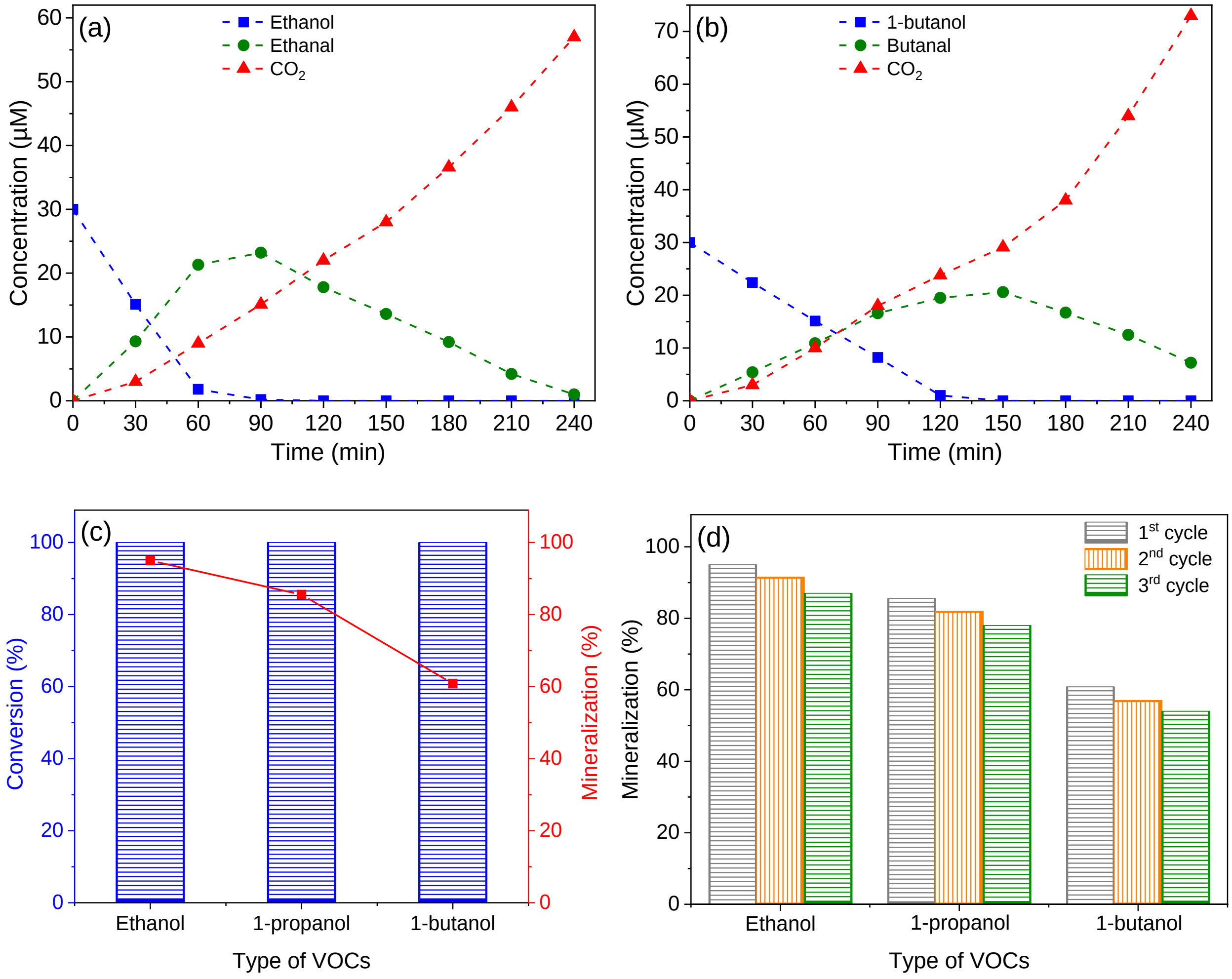

Fig. S7. Photodegradation curves of ethanol (a), 1-butanol (b) and intermediate formation *vs.* illumination time by Ag-Ag_2S/TiO_2/CT; mineralization and conversion rate of different VOCs (c); and reusability of Ag-Ag_2S/TiO_2/CT for photocatalytic mineralization of different VOCs during the three cycles (d).

Table S1. Comparison of photocatalytic activity of some photocatalysts with the present photocatalyst for the degradation of TOD.

Photocatalysts	Concentration and volume of TOD	Light source	Degradation time (h)	Degradation (%)	Reference
Ag-Ag_2S/TiO_2/CT	25 mg/L, 50 ml	100W Xenon lamp	6	97	this work
2% N doped TiO_2 deposited on glass beads	5×10^{-4} M, 25 ml	50 W Halogen lamp	8	45	2
2% Li doped TiO_2 deposited on glass beads	5×10^{-4} M, 25 ml	50 W Halogen lamp	8	55	2
2% Li–N co-doped TiO_2 deposited on glass beads	5×10^{-4} M, 25 ml	50 W Halogen lamp	8	61	2
2% Y–N co-doped TiO_2 deposited on glass beads	5×10^{-4} M, 25 ml	47 W halogen lamp	8	88	3
3% Y–N co-doped TiO_2 deposited on glass beads	5×10^{-4} M, 25 ml	47 W halogen lamp	8	61	3
4% Y–N co-doped TiO_2 deposited on glass beads	5×10^{-4} M, 25 ml	47 W halogen lamp	8	56	3
2% Li doped TiO_2 deposited on glass beads	5×10^{-4} M, 50 ml	54 W Halogen lamp	8	53	4
2% Y doped TiO_2 deposited on glass beads	5×10^{-4} M, 50 ml	54 W Halogen lamp	8	54	4
2% Li–Y co-doped TiO_2 deposited on glass beads	5×10^{-4} M, 50 ml	54 W Halogen lamp	8	63	4
3% Bi–Y co-doped TiO_2 deposited on glass beads	5×10^{-4} M, 50 ml	42 W Halogen lamp	7	50	5

Table S2. *k* and R^2 of different photocatalysts on *o*-toluidine degradation under simulated-sunlight irradiation; (95% confidence intervals).

Photocatalyst name	Kinetic constant (*k*) (min^{-1})	Coefficient of determination (R^2)
Without photocatalyst	9.78×10^{-5}	0.99
TiO_2/CT	0.00121	0.97
Ag-Ag_2S/TiO_2/CT	0.00738	0.94

Table S3. Comparison of photocatalytic activity of some photocatalysts with the present photocatalyst for the degradation of gaseous alcohols.

Photocatalysts	Name of pollutant ; Concentration	Light source	Conversion time (min)	Conversion (%)	Reference
Ag-Ag_2S/TiO_2/CT	Ethanol ; 30 μM	500 W Xenon lamp	~60	~100	this work
Ag-Ag_2S/TiO_2/CT	1-propanol ; 30 μM	500 W Xenon lamp	~90	~100	this work
Ag-Ag_2S/TiO_2/CT	1-butanol ; 30 μM	500 W Xenon lamp	~120	~100	this work
TiO_2 film	Ethanol ; 275 ppm	Iron halogenide lamp (UV)	75	100	6
TiO_2/polymer composite	Ethanol ; 0.36 mM	Sunlight simulator lamp	180	57	7
Ce-TiO_2	Ethanol ; 400 ppm	LED lamp (visible light)	180	23 ± 1	8

TiO_2 Degussa P25	Ethanol ; 118 mg m^{-3}	80 W mercury lamp (UV)	~90	100	9
TiO_2-SiO_2-PMMA	Ethanol ; 198 ppm	Halogenide lamp (UV)	240	100	10
Mn-TiO_2	Ethanol ; 400 ppm	16.8 W LED light	1440	35	11
Cu-TiO_2	Ethanol ; N.A	Four 15-W fluorescent tubes (UV lamp)	360	55	12
Cu-TiO_2	1-propanol ; N.A	Four 15W fluorescent tubes (UV lamp)	360	50	12
TiO_2 Degussa P25	1-propanol ; 400 ppm	TL-D 18W/08 fluorescent tube (UV lamp)	176	62	13
TiO_2	1-butanol ; 1500 mg m^{-3}	Medium pressure mercury lamp	1000	80	14
TiO_2 Degussa P25	1-butanol ;580 ppm	Fluorescent white light (FWL)-lamp (UV lamp)	240	95	15

N.A: Not available

Table S4. Comparison of the photocatalytic *E. coli* inactivation efficiency of Ag-Ag_2S/TiO_2/CT with other reported photocatalysts.

Photocatalysts	Concentration and volume	Light source	Irradiation time (min)	Photocatalytic inactivation efficiency (%)	Reference
Ag-Ag_2S /TiO_2/CT	1 x 10^7cfu/mL, 50 mL	100 W Xenon lamp	60	97	this work
Ag/AgBr/TiO_2 nanotube	1.2 x 10^7cfu/mL, 100 mL	500 W Xe lamp	80	47.3	16
g-C_3N_4/TiO_2	1 x 10^7cfu/mL, 50 mL	300 W Xenon lamp	180	100	17
2%S–TiO_2 and 2%Se–TiO_2	1 x 10^4cfu/mL, 30 mL	Xenon lamp	90	100	18
2%Te–TiO_2	1 x 10^4cfu/mL, 30 mL	Xenon lamp	70	100	18
Ag–TiO_2@rGO	1 x 10^8cfu/mL, 10 mL	250 W Xe lamp	300	71	19
Sr^{2+}/Ag-TiO_2@rGO	1 x 10^8cfu/mL, 10 mL	250 W Xe lamp	300	96	19
MoS_2/TiO_2 nanotube arrays	1 x 10^8cfu/mL, 5 mL	300 W Xenon lamp	150	100	20
CoO_x/Mo-$BiVO_4$	1 x 10^5 cfu/mL, 30 mL	Xenon arc lamp	120	75	21
CuO_x/Mo-$BiVO_4$	1 x 10^5 cfu/mL, 30 mL	Xenon arc lamp	120	86	21
Ag_2O_x/Mo-$BiVO_4$	1 x 10^5 cfu/mL, 30 mL	Xenon arc lamp	120	79	21

References

1 P. Giesz, G. Celichowski, D. Puchowicz, I. Kamińska, J. Grobelny, D. Batory, M. Cieślak, *Cellulose*, 2016, **23**, 2143–2159.

2 Z. Hamden, S. Boufi, D.S. Conceição, A.M. Ferraria, A.M. Botelho do Rego, D.P. Ferreira, L.F. Vieira Ferreira, S. Bouattour, *Appl. Surf. Sci*., 2014, **314**, 910–918.

3 Z. Hamden, D. Conceição, S. Boufi, L.F.Vieira Ferreira, S. Bouattour, *Materials*, 2017, **10**, 600–612.

4 Z. Hamden, D.P. Ferreira, S. Bouattour, *Ceram. Int*., 2014, **40**, 3227–3235.

5 A. Hamdi, A.M. Ferraria, A.M. Botelho do Rego, D.P. Ferreira, D.S.Conceição, L.F. Vieira Ferreira, S. Bouattour, *J. Mol. Catal. A*, 2013, **380**, 34–42.

6 A. Antonello, G. Soliveri, D. Meroni, G. Cappelletti, S. Ardizzone, *Catal. Today*, 2014, **230**, 35–40.

7 L. Janovák, Á. Dernovics, L. Mérai, Á. Deák, D. Sebők, E. Csapó, A. Varga, I. Dékány, C. Janáky, *Chem. Commun*., 2018, **54**, 650–653.

8 H. Khan, M.G. Alalm, M. Lalonde-Lavoie, M.F. Ordonez, M. Sartirana, A. Giordana, G. Cerrato, C.L. Bianchi, D.C. Boffito, *J. Environ. Chem. Eng*., 2021, **9**, 106813.

9 E. Piera, J.A. Ayllón, X. Doménech, J. Peral, *Catal. Today*, 2002, **76**, 259–270.

10 V. Sabatini, L. Rimoldi, L. Tripaldi, D. Meroni, H. Farina, M.A. Ortenzi, S. Ardizzone, *Catalysts*, 2018, **8**, 568.

11 M. Stucchi, D.C. Boffito, E. Pargoletti, G. Cerrato, C.L. Bianchi, G. Cappelletti, *Nanomaterials*, 2018, **8**, 686.

12 J. Araña, A. Peña Alonso, J.M. Doña Rodríguez, J.A. Herrera Melián, O. González Díaz, J. Pérez Peña, *Appl. Catal. B*, 2008, **78**, 355–363.

13 G. Vincent, P.M. Marquaire, O. Zahraa, *J. Hazard. Mater*., 2009, **161**, 1173–1181.

14 F. Benoit-Marquié, U. Wilkenhöner, V. Simon, A.M. Braun, E. Oliveros, M-T. Maurette, *J. Photochem. Photobiol. A*, 2000, **132**, 225–232.

15 J. Kirchnerova, M.-L. Herrera Cohen, C. Guy, D. Klvana, *Appl. Catal. A-Gen*., 2005, **282**, 321–332.

16 Y. Hou, X. Li, Q. Zhao, G. Chen, C.L. Raston, *Environ. Sci. Technol*., 2012, **46**, 4042–4050.

17 G. Li, X. Nie, J. Chen, Q. Jiang, T. An, P.K. Wong, H. Zhang, H. Zhao, H. Yamashita, *Water Res*., 2015, **86**, 17–24.

18 S. Mathew, P. Ganguly, V. Kumaravel, J. Harrison, S.J. Hinder, J. Bartlett, S.C. Pillai, *Mater. Today: Proc*., 2020, **33**, 2458–2464.

19 X. Wei, J. Cao, F. Fang, *RSC Adv*., 2018, **8**, 31822–31829.

20 H. Yan, L. Liu, R. Wang, W. Zhu, X. Ren, L. Luo, X. Zhang, S. Luo, X. Ai, J. Wang, *Chem. Eng. J*., 2020, **401**,126052.

21 V. Manikandan, M.A. Mahadik, I.S. Hwang, W-S. Chae, J. Ryu, J.S. Jang, *ACS Omega*, 2021, **6**, 23901–23912.
